# Balancing Energy Consumption with Hybrid Clustering and Routing Strategy in Wireless Sensor Networks †

**DOI:** 10.3390/s151026583

**Published:** 2015-10-20

**Authors:** Zhezhuang Xu, Liquan Chen, Ting Liu, Lianyang Cao, Cailian Chen

**Affiliations:** 1 School of Electrical Engineering and Automation, Fuzhou University, Fuzhou 350000, China; E-Mails: chenli7uan@163.com (L.C.); n140120058@fzu.edu.cn (T.L.); caolianyangsheep@163.com (L.C.); 2 Fujian Key Laboratory of Industrial Control and Information Security Technology, Science and Technology Department of Fujian Province, Fuzhou 350000, China; 3 School of Electronic, Information and Electrical Engineering, Shanghai Jiao Tong University, Shanghai 200240, China; E-Mail: cailianchen@sjtu.edu.cn

**Keywords:** energy balance, hybrid clustering and routing, energy efficiency, dynamic clustering, data collection, wireless sensor networks

## Abstract

Multi-hop data collection in wireless sensor networks (WSNs) is a challenge issue due to the limited energy resource and transmission range of wireless sensors. The hybrid clustering and routing (HCR) strategy has provided an effective solution, which can generate a connected and efficient cluster-based topology for multi-hop data collection in WSNs. However, it suffers from imbalanced energy consumption, which results in the poor performance of the network lifetime. In this paper, we evaluate the energy consumption of HCR and discover an important result: the imbalanced energy consumption generally appears in gradient k=1, *i.e.*, the nodes that can communicate with the sink directly. Based on this observation, we propose a new protocol called HCR-1, which includes the adaptive relay selection and tunable cost functions to balance the energy consumption. The guideline of setting the parameters in HCR-1 is provided based on simulations. The analytical and numerical results prove that, with minor modification of the topology in gradient k=1, the HCR-1 protocol effectively balances the energy consumption and prolongs the network lifetime.

## 1. Introduction

Wireless sensor networks (WSNs) [1] have been widely used to collect sensing data in various applications, such as industrial control [2,3], environment monitoring [4,5] and transportation systems [6,7]. Since wireless sensors are generally powered by batteries with limited energy, how to improve the efficiency of data collection has attracted considerable attention from different research communities [8,9].

Dynamic clustering has been proven to be an efficient scheme for data collection in WSNs [10,11,12]. In dynamic clustering, sensor nodes are periodically grouped into clusters, which have a leader called the cluster head (CH) and a number of cluster members (CMs). The CH collects the data from the CMs in its cluster, and then, it forwards the aggregated data to the sink. The network operation is divided into rounds, and the cluster-based topology is completely reorganized at the beginning of every round in order to balance the energy consumption of nodes.

Since the original dynamic clustering protocol was proposed in LEACH (Low-Energy Adaptive Clustering Hierarchy) [10], many research works have been proposed to improve the clustering algorithms. EECS (Energy Efficient Clustering Scheme) [13] proposes a novel algorithm for CMs to select reasonable CHs based on their locations. HEED (Hybrid, Energy-Efficient Distributed clustering) [14] proposes an iteration-based algorithm to select well-distributed CHs, and the randosm backoff strategy is adopted in [15,16] to reduce the overhead. Some research works have studied the performance of dynamic clustering in multi-hop data collection. In [17], the authors provide a mathematical framework to determine the optimal number of clusters by minimizing the energy consumption in both single-hop and multi-hop scenarios. The research work in [18] formulates an optimization problem to assign cluster ranges based on the hop count to the sink.

However, to the best of our knowledge, all of the works stated above have not considered the limitation of the transmission range. In [19], the authors argue that the energy efficiency of these works can hardly be achieved with the constraint of network connectivity. Therefore, they propose a hybrid clustering and routing (HCR) protocol that combines the gradient routing with dynamic clustering to generate a connected and efficient inter-cluster topology with limited transmission range. Nevertheless, HCR suffers from the imbalance of energy consumption and hence has poor performance in network lifetime. It motivates us to further exploit the imbalance of energy consumption in HCR and to propose an improved protocol based on HCR in this paper.

In this paper, we provide a detailed analysis of the imbalanced energy consumption in HCR via simulations. The simulation results show that there is over 50% energy left in the network when the first dead node appears. It depicts that the imbalance of energy consumption is severe in HCR. Moreover, we exploit the distribution of the energy consumption in HCR and discover an interesting result: the imbalanced energy consumption generally appears in the nodes that can transmit the data to the sink directly, *i.e.*, the nodes in gradient k=1.

This result motivates us to improve the performance of HCR by balancing the energy consumption in gradient k=1. An intuitive idea is to select reasonable relays for the nodes with low energy in gradient k=1. However, the realization of this idea is a non-trivial issue that has several challenges as follows:

1. Identify the node that requires a relay: Intuitively, the node with low energy should select a relay to reduce its energy consumption. However, due to the periodical cluster reorganization, the residual energy of nodes is variable in different rounds. Thus, it is important to design an adaptive scheme to identify if a node requires the relay.

2. Relay selection: For the node deciding to select a relay, how to select the relay with energy efficiency is an important issue. The relay selection should consider both the efficiency of the transmission path and the load balance between the transmitter and the relay.

3. Taking advantage of HCR: HCR provides an effective solution with low overhead to generate a connected and efficient topology at every round. Therefore, the new algorithm should balance the energy consumption with the advantages of HCR.

To overcome these challenges, we propose the HCR-1 protocol, which focuses on balancing the energy consumption in gradient k=1. In HCR-1, an adaptive energy threshold is proposed to decide whether a node requires a relay, and tunable cost functions are designed to optimize relay selection and cluster formation. Analytical and numerical results show that, with minor modifications of the network topology, HCR-1 can prolong the network lifetime by over 30% compared to that of HCR.

The rest of this paper is organized as follows. Section 2 provides a brief survey of related works. Section 3 introduces the HCR protocol and evaluates the imbalanced energy consumption of HCR. Section 4 describes the details of the HCR-1 protocol. Simulation results are provided in Section 5 to evaluate the performance of HCR-1. Finally, Section 6 provides the conclusion of this paper.

## 2. Related Works

In this section, we will provide a brief introduction about the dynamic clustering and routing protocols that are related to the research works in this paper. Dynamic clustering is firstly proposed in LEACH [10]. The basic idea is to periodically rotate the cluster heads (CHs) and to reorganize the cluster-based topology based on the new set of CHs. In this case, the heavy energy burden of CHs can be dispersed all over the network. Moreover, the CHs can use data aggregation techniques [20,21] to reduce the data volume and the energy consumption for transmitting the data to the sink. Due to the advantages of scalability and energy efficiency, dynamic clustering is considered a promising solution for large-scale data collection in WSNs. Therefore, it has attracted considerable attention from various research communities.

Cluster head selection is a fundamental issue in dynamic clustering. In HEED [14], an iteration-based algorithm is proposed to consider both residual energy and communication cost in cluster head selection. It improves the CHs’ distribution in the network and has better efficiency than LEACH. However, HEED has considerable message overhead due to the iteration in the algorithm. To solve this problem, BSC (Backoff Strategy Clustering) [16] adopts the random backoff scheme to control the process of CH selection. The node with a smaller backoff time has a higher probability to be the CH. BSC can generate well-distributed CHs with low overhead. Different from the distributed algorithms given above, in [22], the harmony search algorithm (HSA) is used to select the CHs with centralized optimization. It is expected to minimize the intra-cluster communication cost and to optimize the energy distribution of the network.

Cluster formation is another important issue in dynamic clustering. EECS [13] proposes a novel cost function for CMs to select proper CHs by considering the difference of the distance between the CH and the sink. The energy consumption of inter-cluster communication is balanced by intra-cluster communication in EECS, and the network lifetime is significantly improved. Different from the design of cluster formation algorithms, some research works have provided mathematical analysis on how to determine the cluster ranges in different scenarios [17,18]. In [17], the authors provide a mathematical framework to determine the optimal number of clusters by minimizing the energy consumption in both single-hop and multi-hop scenarios. EC (Energy-Efficient Clustering) [18] considers the multi-hop data collection scenario and formulates an optimization problem that determines suitable cluster ranges depending on the hop distance to the sink.

The inter-cluster communication has not been considered as an important problem in most research works on dynamic clustering. Many dynamic clustering protocols are developed based on the assumption that the CHs can communicate with the sink directly [10,13,16]. Some research works have considered the multi-hop routing among CHs. For example, HEED [14] uses the greedy routing algorithm for inter-cluster communication, and EC [18] uses a routing algorithm that considers the load balance among CHs. Nonetheless, the routing algorithm and the clustering algorithm are generally considered as two isolated problems in these works.

On the other hand, the research work in [19] proposes an interesting result that the connectivity and efficiency of the inter-cluster topology have tight relations with the cluster head selection. Based on this result, they propose a hybrid clustering and routing (HCR) protocol that combines gradient routing [23,24] with dynamic clustering to generate a connected and efficient inter-cluster topology with limited transmission range. Nevertheless, HCR suffers from the imbalance of energy consumption and, thus, has poor performance in network lifetime.

Compared to related works, the HCR-1 protocol proposed in this paper distinguishes itself from them in two aspects: (1) the balance of energy consumption is considered with the limitation of transmission range. Most related works, such as [14,18], are proposed based on the assumption that all sensor nodes have sufficient transmission power to keep the network connected. However, this assumption can hardly be satisfied in large-scale networks. Therefore, we adopt the hybrid clustering and routing strategy [19] in this paper to generate a connected and efficient topology with limited transmission range; (2) HCR-1 focuses on balancing the energy consumption among the nodes that can transmit the data to the sink directly, *i.e.*, the nodes in gradient k=1. The simulation results prove that the network lifetime can be significantly prolonged by HCR-1 with minor modification of the network topology.

## 3. Preliminaries

In this paper, we consider a multi-hop data collection scenario in a wireless sensor network. The network models are given in Section 3.1. In order to clarify the motivation of this paper, we provide a brief introduction to HCR in Section 3.2 and then analyze the imbalanced energy consumption of HCR in Section 3.3.

### 3.1. Network Model

In this paper, the network is assumed to have the following properties:

(1) A great number of sensor nodes are randomly deployed in a square area. Only one sink is deployed in the area to collect the sensing data from all sensor nodes. All sensor nodes and the sink are stationary once they are deployed.

(2) Sensor nodes have homogeneous capabilities and limited energy, which is powered by batteries. Thus, both energy efficiency and load balance are important issues.

(3) The network is organized into clusters, including cluster heads (CHs) and cluster members (CMs). The CM sends sensing data to its CH directly, and the CH forwards the aggregated data to the sink via multi-hop transmission.

(4) Each node has a maximum transmission range $R_{max}$ that is determined by its hardware capability. Therefore, the connectivity of the cluster-based topology should be considered.

(5) Each node has two transmission ranges to control the network topology: the clustering range $R_{c}$ and the inter-cluster transmission range $R_{t}$. $R_{c}$ determines the size of each cluster, while $R_{t}$ impacts the inter-cluster topology. According to the analysis given in [19], we have $R_{t}=R_{\max}$ and $R_{c}⩽R_{t}$.

(6) The distance between any pair of nodes can be estimated by RSSI (received signal strength indicator) or its geographic location.

In this paper, the energy consumption model is adopted from [10], with the free space channel. The energy consumed for transmitting *l*-bit data over distance *d* is denoted by,

(1) $$E_{tx}\left(l,d\right)=l*\left(E_{elec}+\epsilon_{fs}*d^{2}\right)$$

and the energy consumed for receiving *l*-bit data is:

(2) $$E_{rx}\left(l\right)=l*E_{elec}$$

where $E_{elec}$ and $\epsilon_{fs}$ are the parameters of transmission and reception circuits. Their values are set as $E_{elec}$ = 50 nJ/bit and $\epsilon_{fs}$ = 10 pJ/bit/m^2^. Besides, the energy for data aggregation is set as $E_{DA}$ = 5 nJ/bit/signal.

### 3.2. HCR Protocol

The HCR protocol [19] consists of three algorithms: gradient field establishment, cluster head selection and routing discovery. To clarify the statement, all the variables used in HCR and HCR-1 are summarized in Table 1.

#### 3.2.1. Gradient Field Establishment

In gradient field establishment, all nodes obtain their gradient *k* by flooding an advertisement (ADV) message all over the network. At first, the sink sets its gradient k=0 and broadcasts the ADV message, which contains $k_{ADV}=0$ within range $R_{t}$. The node that receives this ADV message will set its gradient as $k=k_{ADV}+1=1$ and then broadcast the ADV message with $k_{ADV}=1$ in range $R_{t}$. The flooding process continues until all nodes obtain their gradients. If a node receives more than one ADV messages, it only recognizes the first ADV message and ignores all of the following ADV messages.

**Table 1:** List of variables.

Symbol	Description
$R_{c}$	Clustering range
$R_{t}$	Inter-cluster transmitting range
*N*	Number of nodes
*k*	Gradient, which equals the minimum hop count to the sink
R(j)	Ring, the set of nodes with the same gradient *j*
$d_{toEdge}$	The estimated distance to the edge of the gradient field
d(i,j)	Distance between node *i* and *j*
d(i,0)	Distance between node *i* and the sink
$T_{b}$	Backoff timer
$E_{max}$	Initial energy of nodes
$E_{r}$	Residual energy of nodes
$E_{ave}$	Average residual energy of nodes

It is worth noting that the gradient *k* depicts the minimum hop that counts from the node to the sink. Besides, the gradient field establishment updates only once at the beginning of the network operation. Thus, it does not increase the complexity of the protocol.

To clarify the descriptions, we have the following definitions:

**Definition 1.** *(Gradient) Given the transmission range* $R_{t}$, *the gradient of node i, which is denoted as* k(i), *is the minimum hop count by forwarding a packet from node i to the sink with range* $R_{t}$.

**Definition 2.** *(Ring) The set of nodes that have the same gradient j is defined as Ring j, which is denoted by,*

(3) R(j)={i:k(i)=j,i∈S}

*where* S *is the set of all sensor nodes*.

**Definition 3.** *(Distance) Given node i with gradient k, the distance from the node i to the sink is denoted as* d(i,0). *The shortest distance from the node i to the edge of* R(k-1) *can be formulated as* $d_{toEdge}$,

(4) $$d_{toEdge}=d\left(i,0\right)-\left(k-1\right)\times R_{t}$$

#### 3.2.2. Cluster Head Selection

After the gradient field is established, the gradient information *k* can be exchanged distributively among nodes for making the decisions about the cluster head selection and routing discovery. The cluster head selection algorithm in HCR is driven by the random backoff scheme [16]. The node decides whether to be the CH according to its back-off timer $T_{b}$ and received ADV messages. The timer $T_{b}$ is determined by the gradient *k* and the residual energy $E_{r}$,

(5) $$T_{b}=\left(K-k\right)\times T_{slot}+\left(\frac{E_{max}-E_{r}}{E_{max}}\right)\times T_{slot}$$

where *K* is the maximum value of the gradient *k* and $E_{max}$ is the initial energy of every node. According to Equation (5), the cluster head selection process is divided into *K* slots, whose length is $T_{slot}$, and the set of CHs in R(k) is selected in the (K-k) slot. Besides, the nodes with more residual energy have smaller $T_{b}$ in the same R(k), which means a higher probability to become the CH.

Another factor that impacts the cluster head selection is the ADV message broadcast by the CH. When a node is determined to be the CH, it should broadcast the ADV message for clustering (AC) in $R_{c}$ and the ADV message for routing (AR) in $R_{t}$. Both AC and AR carry its ID (identifier), gradient information (*k*), residual energy ($E_{r}$) and NEXT flag which denotes if it has found its next hop relay. According to the received messages, each node updates the data lists which are defined as follows:

(1) JOIN list: record the cluster head candidates.

(2) SERV list: record the requests for a relay from the cluster heads with larger gradient *k*.

When a node receives AC message, which means that there is a reasonable CH with more residual energy nearby, the node will record the sender information in the JOIN list. On the other hand, when a node receives AR message with $k_{AR}>k$, it will check the NEXT flag in the AR message. If NEXT = 0, which means that the sender CH has not found a relay, the node will record the sender information in the SERV list and serve as a candidate relay for the sender CH.

When $T_{b}$ terminates, the node checks its JOIN and SERV lists. The node will turn out to be a CH if one of the following conditions is satisfied:

(1) The JOIN list is empty, which means that the node has the smallest backoff timer in its one-hop neighbors.

(2) The SERV list is not empty, which means that the node is required to be a relay for another CH and ensures the network is connected.

Otherwise, the node will become a CM.

#### 3.2.3. Routing Discovery

The goal of routing discovery is to find a relay for every node. According to Equation (5), the CH in R(k) could not find its relay in the (K-k)-th slot. Hence, the first AR message is broadcast with NEXT = 0. Then, the CH waits for its next-hop to appear. When the CH receives the AR message with $k_{AR}<k$, the CH selects the sender as its relay and broadcasts the second AR message with NEXT = 1 to announce that it has found a relay. For the CH in R(1), its relay is destined to be the sink. For the node that decides to be a CM, it chooses its CH which has the highest residual energy in the JOIN list. Then it starts to transmit sensing data to its CH.

### 3.3. Imbalanced Energy Consumption in HCR

**Figure 1:**
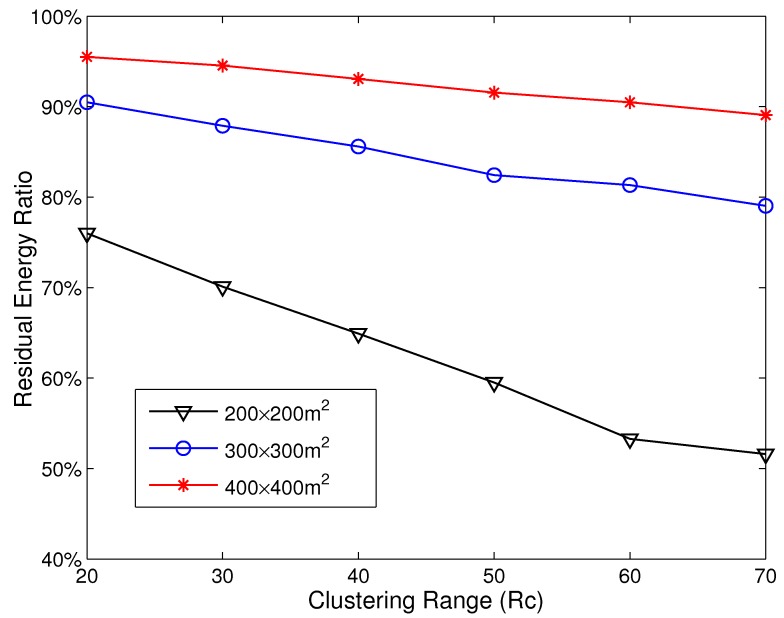
The residual energy ratio with different $R_{c}$.

In this section, we use simulations to exploit the imbalance of energy consumption in HCR. The residual energy ratio is used to demonstrate the degree of energy imbalance, which is defined as the ratio of residual energy to initial energy when the first dead node appears. At first, simulations are implemented in the network with its area varying from 200 × 200 m^2^ to 400 × 400 m^2^, and the node density is set as 0.01 node/m^2^. The sink resides at coordinate (0,0). $R_{t}$ is set as 70 m, and $R_{c}$ varies from 20 m to 70 m. The simulation results are given in Figure 1.

As shown in Figure 1, the residual energy ratio increases with the growth of network area and decreases as the $R_{c}$ enlarges. When the side length of the network is 400 m and $R_{c}=70$ m, the residual energy ratio is near to 90%. When the side length is 200 m and $R_{c}=70$ m, there is still over 50% energy left in the network when the first dead node appears. This indicates that there is a severe imbalance of energy consumption in HCR.

**Figure 2:**
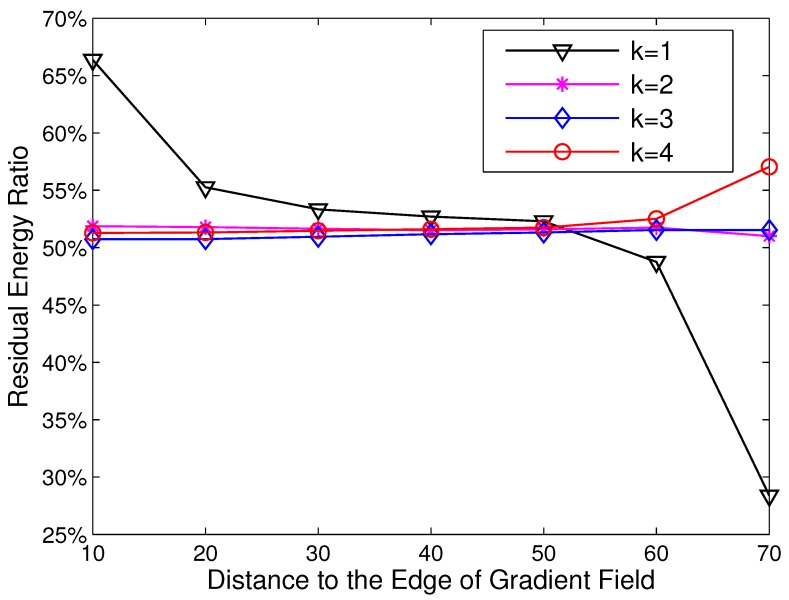
The residual energy ratio with different node locations.

To further exploit the reason for the imbalanced energy consumption, we decided to study the distribution of residual energy with different *k* and $d_{toEdge}$ (the definitions are given in Section 3.2.1). In this simulation, the side length of the network is set to 200 m, and the clustering range $R_{c}$ is set as 70 m. The results are given in Figure 2.

Figure 2 demonstrates an interesting result that the imbalance of energy consumption in HCR almost appears in R(1). In R(1), the residual energy ratio varies from 67% to 28% with the growth of $d_{toEdge}$. While in other rings, the residual energy ratio keeps stable at about 52% and has little relation to $d_{toEdge}$. This is caused by the gradient routing strategy adopted in HCR. Each CH has to forward its data to the CH, which has a smaller gradient. For the CHs with gradient k⩾2, their transmission ranges are relatively stable based on the gradient field. However, all of the CHs with gradient k=1 have to transmit their data to the sink directly. Due to the difference in node location, the transmission range in R(1) is variable, and that results in the imbalance of energy consumption.

## 4. HCR-1 Protocol Description

Based on the analysis given in Section 3.3, the imbalanced energy consumption in R(1) is the bottleneck of HCR, which limits the network lifetime. In this paper, we propose an improved protocol, called HCR-1, which aims to balance the energy consumption in R(1).

The framework of the HCR-1 protocol is built based on HCR, such that the HCR-1 can inherit the advantages of HCR directly. The gradient field establishment algorithm is the same as HCR, and the random backoff scheme is used in conjunction with gradient information exchange to generate a connected and efficient network topology with limited transmission range $R_{max}$. According to the analysis given in Section 3.3, the energy consumption of nodes with k⩾2 is well balanced. Thus, the clustering and routing algorithms for nodes with k⩾2 are the same as those in HCR. These algorithms have been introduced in Section 3.2. Please refer to it for the details.

**Figure 3:**
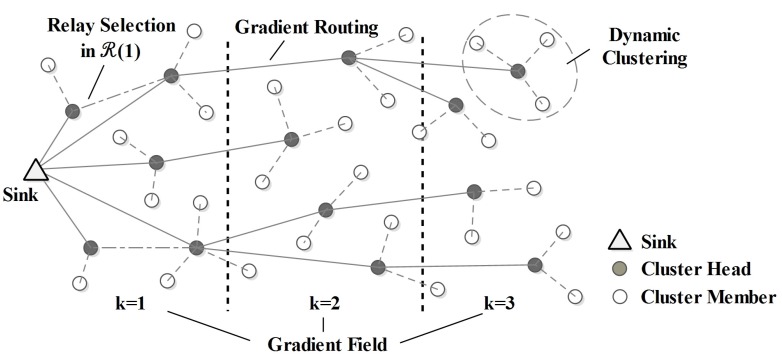
The network topology of HCR-1.

Different from HCR, HCR-1 proposes a set of algorithms to balance the energy consumption in R(1). The basic idea of HCR-1 is to select reasonable relays for the nodes in R(1), such that the traffic load can be well balanced. An adaptive energy threshold is used for the node to decide whether it requires a relay, and the cost functions are designed to select relays. Besides, the clustering and routing algorithms in R(1) are redesigned to generated well-balanced clusters and routes. An example of the network topology generated by HCR-1 is illustrated in Figure 3. The details of HCR-1 are given as follows.

### 4.1. Cluster Head Selection in R(1)

In HCR-1, the CHs in R(1) are selected with two goals:

(1) To guarantee the network connectivity, *i.e.*, to serve as the relay for the CHs in R(2);

(2) To balance the energy consumption in R(1).

For the network connectivity, the cluster head selection is driven by the gradient information exchange and random backoff strategy. This means that the status of a flat node has a tight relation to its backoff timer $T_{b}$ and received messages.

To balance the energy consumption, the basic idea of HCR-1 is to select a reasonable relay for the CH with low energy, such that its energy consumption can be reduced. To realize this idea, there are two problems that need to be solved: (1) how to identify a CH that requires a relay; (2) how to identify a node is able to be a relay candidate. In HCR-1, these problems are solved in the cluster head selection algorithm. Since the nodes in R(1) can communicate with the sink directly, we use the average residual energy to classify the nodes. At first, the definition of average residual energy is given as follows.

**Definition 4.** *(Average residual energy) Assume that the set of nodes* R(j) *includes node* i=1,2,3…N, *and the residual energy of node i is denoted as* $E_{r}\left(i\right)$. *The average residual energy in* R(j) *is denoted as* $E_{ave}\left(j\right)$, *which is formulated by,*

(6) $$E_{ave}\left(j\right)=\frac{\sum_{i=1}^{N}E_{r}\left(i\right)}{N},i\in R\left(j\right)$$

At the end of each round, every node in R(1) is required to forward its residual energy $E_{r}$ with its data packet to the sink, such that the sink can calculate the $E_{ave}\left(1\right)$ by Equation (6). At the beginning of the next round, the sink broadcasts an ADV message with $E_{ave}\left(1\right)$ in range $R_{t}$, such that every node in R(1) can receive it.

Once the node in R(1) receives the message with $E_{ave}\left(1\right)$, it compares its residual energy $E_{r}$ to $E_{ave}\left(1\right)$. If $E_{r}>E_{ave}\left(1\right)$, it will set its NEXT flag (NEXT = 1). Otherwise, it will clear its NEXT flag (NEXT = 0). In R(1), the NEXT flag is used to classify the nodes. The node with NEXT = 1 has sufficient energy to communicate with the sink directly, and it can serve as a relay for other nodes in R(1). On the other hand, the node with NEXT = 0 has to select a reasonable relay to reduce its energy consumption. It is worth noting that the function of the NEXT flag in R(1) is different from that in other rings. Please refer to Section 3.2.2 for the details. The NEXT flag will be exchanged among nodes during the cluster head selection.

After determining its NEXT flag, the node sets up a backoff timer $T_{b}\left(i\right)$ according to Equation (5). Then, it listens to the channel for incoming messages. the messages are only broadcast by CHs. There are two kinds of messages: ADV message for clustering (AC) and ADV message for routing (AR). Both AC and AR messages carry the information of the CH, including its ID, gradient *k*, residual energy ($E_{r}$), NEXT flag and its distance to sink d(i,0). This information will be used in the following algorithms.

When a node *i* receives an AC message from a CH *j*, which means a proper CH is located within its clustering range $R_{c}$, it will record the CH *j* in the JOIN list. However, the AR message could be sent by CHs in R(1) and R(2). Thus, the node that receives AR messages should deal with them in different ways.

When the node *i* receives AR message from a CH *j* with k(j)=2, it will check the NEXT flag in this AR message. If NEXT(j) = 0, which means the CH *j* in R(2) has not found a forwarding relay, the node will become its relay candidate and record its information in the SERV list. If NEXT(j) = 1 and the CH *j* has already been recorded in the SERV list, which means the CH *j* in R(2) has found its relay, the node will delete the information of CH *j* from the SERV list.

On the other hand, when the node *i* receives AR message from a CH *j* with k(j)=1, it will check the NEXT flag in the AR message and estimate its distance to the CH *j*, denoted as d(i,j). If NEXT(j) = 1 and d(i,j)<d(i,0), the node will record the information of CH *j* in the RELAY list. The RELAY list saves the information of CHs that are its relay candidates. The conditions of NEXT(j) = 1 and d(i,j)<d(i,0) ensure that the relay candidate has sufficient energy and that it is closer than the sink.

When the backoff timer $T_{b}\left(i\right)$ terminates, the node firstly checks its NEXT flag. If NEXT = 1, the node will become a CH and communicate with the sink directly. If NEXT = 0, it will further check its JOIN and SERV list. If SERV is not empty or JOIN is empty, this means that the node is required to serve as a relay for the CH in R(2) or it has the highest residual energy within its clustering range $R_{c}$. In this case, the node will become a CH to ensure the network connectivity, and it will select a relay from its RELAY list to reduce its energy consumption.

Otherwise, if a node does not satisfy the conditions given above, which means that this node does not impact the network connectivity and has relatively low residual energy, it will become a CM.

### 4.2. Routing Discovery in R(1)

After the cluster head selection, each CH needs to find its next-hop node in the routing algorithm. The CHs in R(1) are divided into two sets according to their residual energy: CHs with NEXT = 1 and CHs with NEXT = 0. As stated in Section 4.1, the CHs with NEXT = 1 are determined to communicate with the sink directly, since they are sufficient in residual energy. On the other hand, the CHs with NEXT = 0 should select a closer relay to reduce the energy consumption. In HCR-1, a routing discovery algorithm is proposed for the CHs with NEXT = 0 to select a reasonable relay from the RELAY list.

According to the model given in Section 3.1, the energy consumption is tightly relevant to the number of data packets and the transmission distance. To achieve energy balance, the relay selection should consider the energy consumption of both the sender CH and the receiver CH. Therefore, given a CH *j* and its relay candidates in the RELAY list, the CH *j* uses the routing cost function to select its relay.

(7) $$C_{route}\left(i,j\right)=\alpha \times d^{2}\left(i,j\right)+\left(1-\alpha \right)\times d^{2}\left(i,0\right),i\in RELAY$$

where the CH *i* is a CH in the RELAY list and d(i,j) is the distance between CH *i* and CH *j*. d(i,0) is the distance between CH *i* and the sink. The *α* is the routing factor for the tradeoff of energy consumption between CH *i* and CH *j*. The CH *i* with minimum $C_{route}\left(i,j\right)$ will be selected as the relay of CH *j*. The value of the routing factor *α* has great impacts on the network lifetime, which will be discussed in Section 5.1.

It is worth noting a special condition that the CH with NEXT = 0 has an empty RELAY list. In this case, the CH has to transmit its data to the sink directly.

### 4.3. Cluster Formation in R(1)

The CMs in R(1) have to decide which cluster to join with the cluster formation algorithm. In related works, the CM selects its CH with the consideration of the distance or the residual energy separately. For example, in [10], the CM chooses its CH with minimum transmission range. In [19], the CM selects its CH with the highest residual energy within the transmission range. However, pursuing the energy efficiency of CM in cluster formation may increase the traffic load of CH. On the other hand, considering the residual energy of CH in cluster formation may reduce the energy efficiency of CMs.

Therefore, in HCR-1, we propose a cluster formation algorithm with joint consideration of the transmission efficiency of CM and the residual energy of CH. Given a CM *j* and a set of CHs in its JOIN list, the CM *j* uses the clustering cost function to select its CH.

(8) $$C_{form}\left(i,j\right)=\beta \times \frac{d(i,j)}{R_{c}}+\left(1-\beta \right)\times \left(\frac{E_{MAX}-E_{r}\left(i\right)}{E_{MAX}}\right),i\in JOIN$$

where d(i,j) is the distance between CH *i* and CM *j* and $E_{r}\left(i\right)$ is the residual energy of the CH *i*. $R_{c}$ is the clustering range, and $E_{MAX}$ is the initial energy of each node. The parameter *β* is the clustering factor for the tradeoff between CM and CH.

The CH *i* with minimum $C_{form}\left(i,j\right)$ will be selected as the CH for CM *j*. The impacts of *β* on the network lifetime will be evaluated in Section 5.1. **Algorithm 1** Clustering and routing algorithm in R(1).
1:Node *i* receives $E_{ave}\left(1\right)$ from the sink;2:**if** $E_{r}\left(i\right)⩾E_{ave}\left(1\right)$ **then**3:  NEXT(i)←1;4:**else**5:  NEXT(i)←0;6:**end if**7:Sets up random backoff timer $T_{b}\left(i\right)$ (Equation (5));8:**repeat**9:  **if** Receives AC message from CH *j* **then**10:      Records CH *j* to JOIN list;11:  **end** **if**12:  **if** Receives AR message from CH *j* AND k(j)=2 **then**13:      **if** NEXT(j)=0 **then**14:            Records CH *j* to SERV list;15:      **else** **if** NEXT(j)=1 AND CH *j* is in SERV list **then**16:            Deletes CH *j* from SERV list;17:      **end** **if**18:  **else** **if** Receives AR message from CH *j* AND k(j)=1 **then**19:      **if** NEXT(j)=1 AND d(i,j)<d(i,0) **then**20:            Records CH *j* to RELAY list;21:      **end** **if**22:  **end** **if**23:**until** $T_{b}$ terminates24:**if** NEXT(i)=1 **then**25:  status←CH;26:  Forwards the data to the sink directly;27:  Broadcasts AC and AR message;28:**else** **if** SERV is *not* empty *or* JOIN is empty **then**29:  status←CH;30:  **if** RELAY is empty **then**31:      Forwards the data to the sink directly;32:  **else**33:      Selects the relay with minimum $C_{route}$ from RELAY list (Equation (7));34:  **end** **if**35:  Broadcasts AC and AR message;36:**else**37:  status←CM;38:  Selects its CH with minimum $C_{form}$ from JOIN list (Equation (8));39:**end** **if**

### 4.4. Protocol Properties

Based on the algorithms given above, HCR-1 has the following properties.

**Lemma 1**. *In* R(1), *when a node decides to be a CH, it can immediately select the sink or a CH with NEXT=1 as its next-hop node*.

**Proof**. The CHs in R(1) can be classified by the NEXT flag. For the CH with NEXT = 1, its next-hop is destined to be the sink. For the CH with NEXT = 0, it has less residual energy than CH with NEXT = 1. According to Equation (5), the backoff timer $T_{b}$ in the same R(1) lasts longer with lower residual energy. Thus, the CHs with NEXT = 1 have to broadcast their AR messages before the CHs with NEXT = 0. The CH with NEXT = 0 has recorded all of its relay candidates in the RELAY list before its backoff timer $T_{b}$ terminates. Therefore, if the RELAY list is not empty, it can select one proper relay based on the routing cost function as soon as it becomes a CH. However, if the RELAY list is empty, the CH with NEXT = 0 selects the sink as its next hop. ☐

**Lemma 2**. *In* R(1), *when a node decides to be a CM, it can immediately select a proper CH as its next-hop node*.

**Proof**. According to the cluster head selection algorithm in R(1), that the JOIN list is not empty is the necessary condition of becoming a CM. This means that there are CHs within its clustering range $R_{c}$. Therefore, the node in R(1) could select the proper CH in its JOIN list based on the clustering cost function as soon as it becomes a CM. ☐

Lemmas 1 and 2 prove that the HCR-1 protocol completes the clustering and routing simultaneously. Based on these results, the network topology of HCR-1 has the following properties.

**Theorem 1**. *In HCR-1, the CH in* R(1) *transmits its data to the sink in one to two hops*.

**Proof**. According to Lemma 1, the CH with NEXT = 1 transmits its data to the sink directly, and the CH with NEXT = 0 forwards its data to the CH with NEXT = 1. This means that the CH with NEXT = 1 transmits data to the sink in one hop, and the CH with NEXT = 0 transmits data to the sink in two hops. ☐

**Theorem 2**. *In HCR-1, the CM in* R(1) *transmits its data to the sink in two to four hops*.

**Proof**. Since the clustering range $R_{c}$ is constrained by $R_{c}⩾R_{t}$, the CHs in the JOIN list of CM in R(1) are only in R(1) or R(2). In the best case, the CM in R(1) selects its CH in R(1) with NEXT = 1. Thus, the hop count from the CM to the sink is minimized to two hops based on Theorem 1. In the worst case, the CM selects its CH *j* in R(2), and the next-hop node of the CH *j* is a CH in R(1) with NEXT = 0. According to Theorem 1, the maximum hop count from the CH in R(1) is two, which makes the maximum hop count three from the CH in R(2) to the sink. In this case, the data transmission from a CM, who selects its CH in R(2), to the sink is maximized to four hops. To summarize, the CM R(1) transmits its data to the sink in two to four hops. ☐

**Theorem 3**. *The HCR-1 protocol generates a connected network topology*.

**Proof**. According to Theorem 2 and Theorem 1, the nodes in R(1) transmit data to the sink in limited hops. On the other hand, the algorithms for nodes with k⩾2 are the same as HCR. Combined with the analytical results given in [19], the network connectivity of k⩾2 is guaranteed. Therefore, the network is ensured to be connected. ☐

**Theorem 4**. *The HCR-1 protocol is completely distributed*.

**Proof**. A node changes its status and selects its next-hop node according to received ADV messages, its backoff timer $T_{b}$ and residual energy $E_{r}$. The ADV messages are broadcast by the CHs within its range $R_{c}$ and $R_{t}$. The $T_{b}$ and $E_{r}$ are determined by the local information. Therefore, the HCR-1 protocol is completely distributed. ☐

## 5. Simulation Results

In this section, we study how the parameter settings impact the performance of HCR-1 and then compare the performance of HCR-1 with that of HCR. The network lifetime and residual energy ratio are used to evaluate the load balance of the protocols. In this paper, the network lifetime is defined as the operating rounds when the first dead node (FND) appears [10], and the residual energy ratio is the ratio of residual energy to initial energy at the end of the network lifetime. In addition, the hop count from node to sink is used to evaluate the network topology. The network is assumed to be located in a square area, and the sink resides at the corner coordinate (0,0). Both $R_{max}$ and $R_{t}$ are set to 70 m. The other parameters used in the simulations are summarized in Table 2.

**Table 2:** Simulation parameters.

Type	Parameter	Value
Application	Initial energy	2 J
	Data packet size (*l*)	125 Bytes
	Sink location	(0,0)
	Round	20 TDMA frames
Radio model	$E_{elec}$	50 nJ/bit
	$\epsilon_{fs}$	10 pJ/bit/m^2^
	$E_{DA}$	5 nJ/bit/signal

### 5.1. Parameter Settings

In this section, we investigate the impact of routing factor *α* and clustering factor *β* on the network lifetime and provide a guideline for setting these parameters. In the default setting of the simulation, there are 400 nodes randomly deployed in a 200 × 200 m^2^ area. The clustering range $R_{c}$ is set as 70 m.

**Figure 4:**
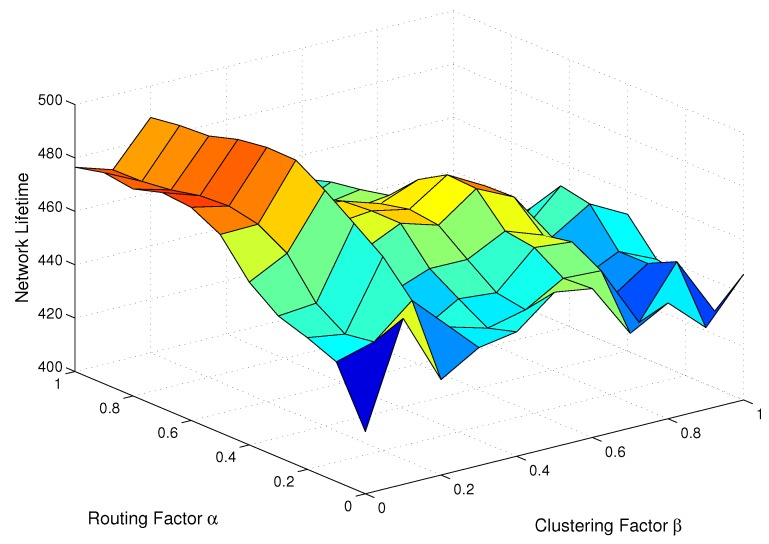
Network lifetime with different routing factor *α* and clustering factor *β*.

At first, we evaluate the network lifetime when *α* and *β* vary from 0 to 1. As shown in Figure 4, the optimal value of *β* is 0.2, and it is irrelevant with respect to the value of *α*. According to Equation (8), the *β* determines how to select the CH for each CM, and it will impact the scale of clusters in R(1). This indicates that the setting of β=0.2 is optimal to reach the load balance of all clusters. Therefore, the *β* is set as 0.2 in the rest of the simulations.

On the other hand, the optimal *α* varies from 0.5 to 1 with different values of *β*. Nevertheless, since the optimal *β* is fixed at 0.2, the optimal setting of the parameters can be obtained by α=0.6 and β=0.2 from Figure 4. In HCR-1, the routing factor *α* plays an important role in selecting reasonable relays. Thus, in the rest of this section, we investigate how to set the optimal value of *α* in different network scenarios.

**Figure 5:**
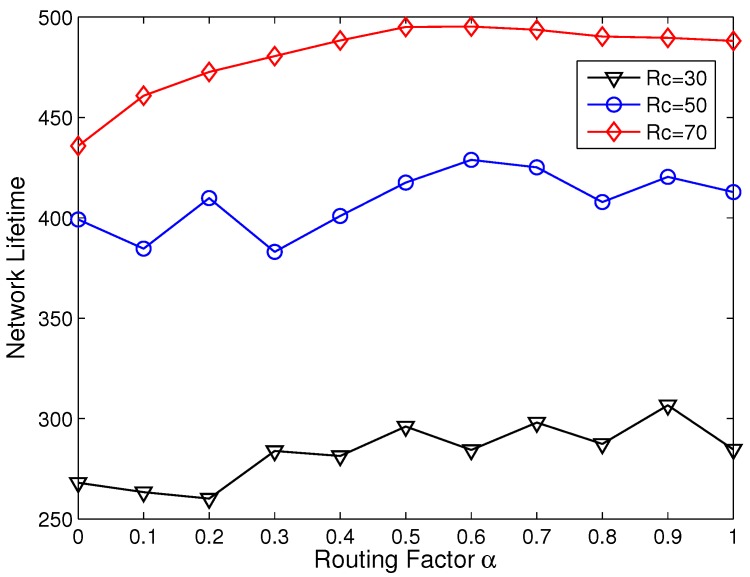
Network lifetime with different routing factor *α* and clustering range $R_{c}$.

Figure 5 shows the relations between *α* and network lifetime when $R_{c}$ grows from 30 m to 70 m. The optimal *α* varies from 0.6 to 0.9 with respect to different $R_{c}$, and the optimal *α* gets larger with smaller $R_{c}$. Generally, when $R_{c}$ gets smaller, the node will receive less AC messages before its backoff timer $T_{b}$ terminates. Thus, it will increase the number of CHs due to the growth of the probability that the JOIN list is empty. In this case, selecting a closer relay is helpful to improve the energy efficiency. This is the reason that the optimal *α* gets larger with smaller $R_{c}$.

The optimal network lifetime is approached with $R_{c}=70$ m and α=0.6. The clustering range $R_{c}$ has been proven to have great impacts on the network lifetime [19,25]. Figure 5 shows that, with the constraint of $R_{max}=R_{t}=70$ m to keep the network connectivity, the growth of $R_{c}$ is helpful to reduce the inter-cluster traffic load and prolong the network lifetime.

**Figure 6:**
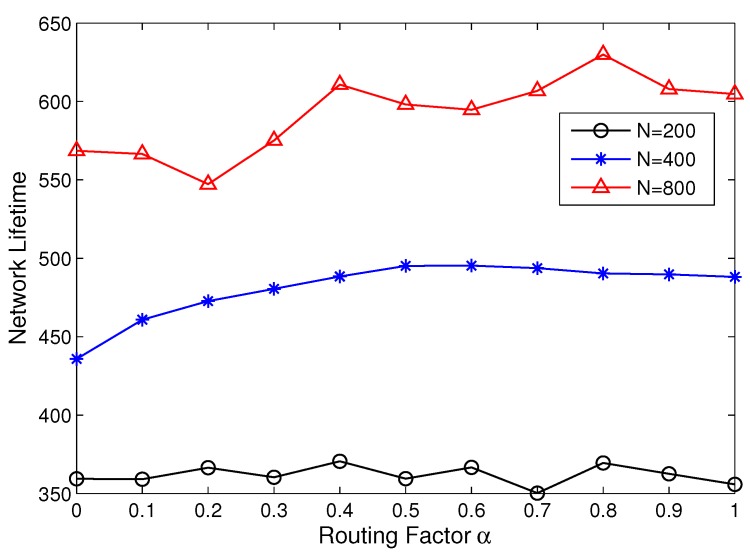
Network lifetime with different routing factor *α* and node densities.

Then, we study the optimal *α* when the number of nodes *N* is set as 200, 400 and 800. The network area is fixed at 200 × 200 m^2^, and the clustering range $R_{c}=70$ m. Figure 6 shows that the network lifetime becomes longer with a larger number of nodes. This is because the distance between nodes is shorter, and there are more nodes that can be used to balance the energy consumption. The optimal *α* is 0.6 when N=400, while the optimal *α* is 0.8 with N=800. This is because the number of relay candidates in R(1) increases with more neighbor nodes. Therefore, the CHs with low energy tend to select a closer relay to avoid early failure, which makes the optimal *α* larger.

It is worth noting that the *α* does not have an optimum value when N=200. When the nodes are not dense enough, there are few CHs that can serve as relays. In this case, the inter-cluster cost function has little effect on relay selection.

In the rest of the simulations, the parameters of *α* and *β* are set to achieve the maximum network lifetime with different network scenarios.

### 5.2. Energy Consumption Analysis

The design goal of HCR-1 is to balance the energy consumption of nodes in R(1). To verify the effectiveness of HCR-1, we firstly explore the distribution of the residual energy ratio and compare to the result of HCR given in Section 3.3. For fairness, the simulation setting is the same as that in Section 3.3: there are 400 nodes randomly deployed in a 200 × 200 m^2^ network area, and the clustering range $R_{c}$ is set as 70 m.

**Figure 7:**
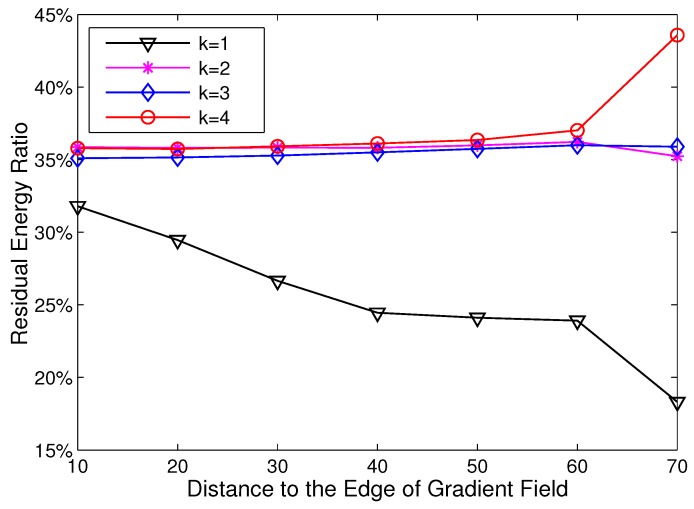
Residual energy ratio with different node locations.

The results are illustrated in Figure 7 with different gradient *k* and $d_{toEdge}$ (Equation (4)). Compared to the results of HCR given in Figure 2, in HCR-1, the maximum residual energy ratio in R(1) decreases from 67% to 32%, and the variance of the residual energy ratio in R(1) is reduced from 39% to 14%. The result proves that the imbalance of the energy consumption in R(1) is effectively alleviated by HCR-1.

**Figure 8:**
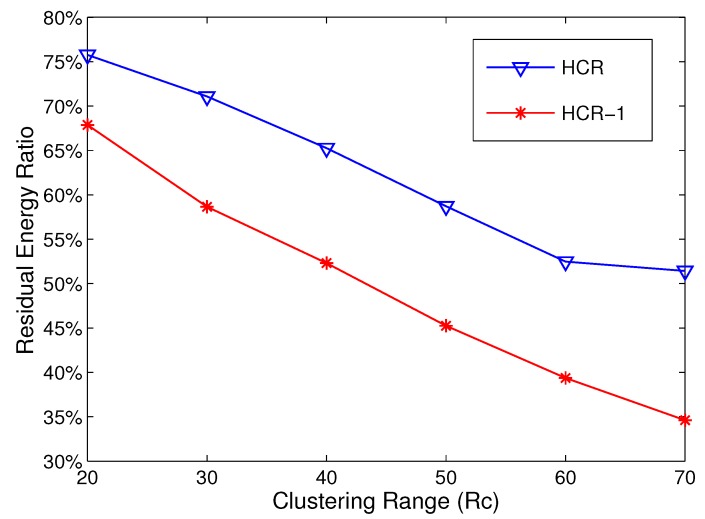
Residual energy ratio comparison with different $R_{c}$.

Moreover, as shown in Figure 7, the residual energy ratio in gradient k⩾2 also decreases in HCR-1. To clarify the improvement, we compare the residual energy ratio of HCR-1 to that of HCR when $R_{c}$ varies from 20 m to 70 m and the network area is fixed at 200 × 200 m^2^. As shown in Figure 8, the residual energy ratio of HCR-1 is smaller than that of HCR, and the improvement gets larger as the $R_{c}$ increases. When $R_{c}=70$ m, the residual energy ratio reduces from 52% in HCR to 35% in HCR-1. The result indicates that, balancing the energy consumption of the nodes in R(1), which is the bottleneck of the whole network, can greatly improve the energy efficiency of the whole network and prolong the network lifetime. Therefore, in the next section, the simulation results will be given to demonstrate the improvement of HCR-1 on the network lifetime.

### 5.3. Network Lifetime Comparison

**Figure 9:**
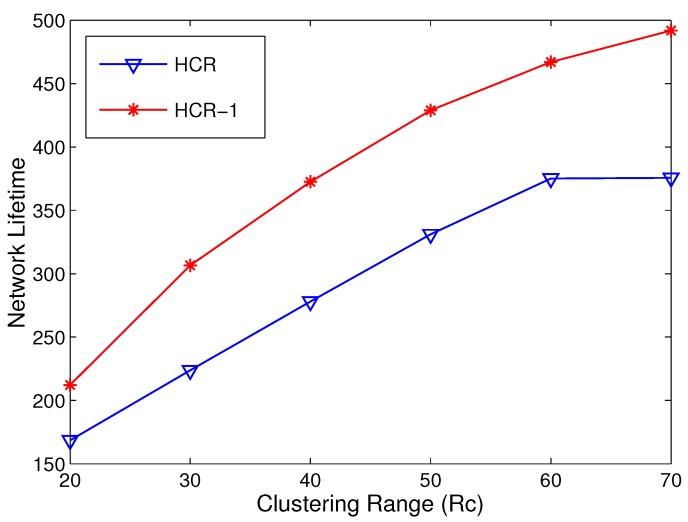
Network lifetime comparison with different $R_{c}$.

In this section, we compare the network lifetime between HCR and HCR-1 with different network scenarios. At first, the simulations are implemented to study the network lifetime with $R_{c}$ in the range [20 m, 70 m]. The simulation is run in the network with 400 nodes randomly deployed in an area of 200 × 200 m^2^. As shown in Figure 9, in both HCR and HCR-1, the network lifetime gets longer with the growth of $R_{c}$, and the network lifetime reaches the maximum when $R_{c}=R_{t}=70$ m. Compared to HCR, the improvement of the network lifetime of HCR-1 is over 30% when $R_{c}=70$ m. The main reason is that the energy consumption in R(1) is effectively balanced by adding relays for the CHs with low residual energy.

**Figure 10:**
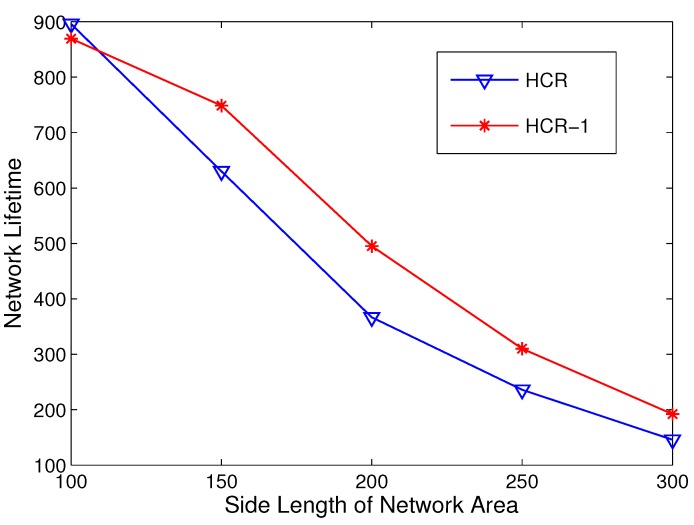
Network lifetime comparison with different network areas.

Then, we study the network lifetime in different network areas. The simulations are carried out with the side length of network area varying from 100 m to 300 m. The node density is fixed at 0.01 node/m^2^, and the $R_{c}$ is set to 70 m. The results are given in Figure 10.

Generally, HCR-1 performs better than HCR when the network area is larger than 100 × 100 m^2^, since the imbalance of energy consumption in R(1) is alleviated by the relay candidate scheme in HCR-1. Nevertheless, HCR-1 does not have better performance in a small area, such as 100 × 100 m^2^. This is because the traffic load accumulated by CHs is not severe in a small-scale network. On the other hand, the advantage of HCR-1 decreases when the network area grows larger than 250 × 250 m^2^. The reason is that HCR-1 focuses on balancing the energy consumption in R(1), but can hardly solve the energy imbalance in other gradients. The energy imbalance among different gradients gets worse with the growth of the network area. Therefore, it reduces the benefits of HCR-1.

**Figure 11:**
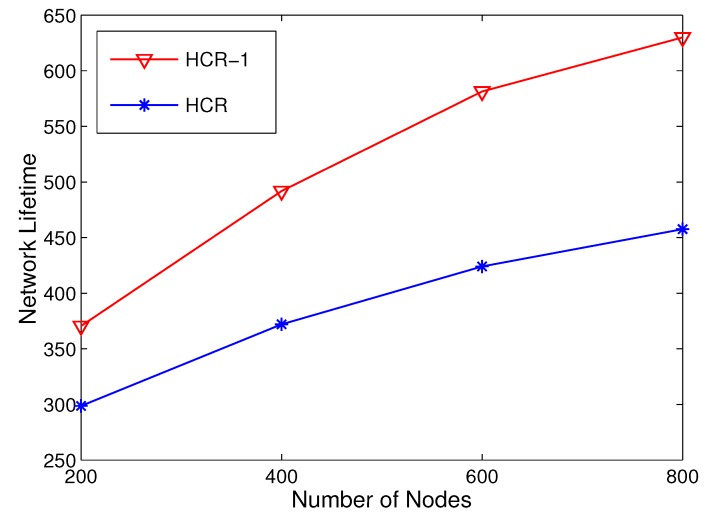
Network lifetime comparison with different node densities.

At last, we study the impacts of node density on network lifetime. Considering a fixed network area of 200 × 200 m^2^, the number of nodes is set as 200, 400, 600 and 800. As shown in Figure 11, the network with higher node density has longer network lifetime due to more energy resources. Moreover, in HCR-1, the improvements of network lifetime become larger when the node density increases. This is because when the number of nodes grows, there are more CHs that can serve as relays for the CH with low energy. In addition, the relay selection can be more reasonable with more candidates. Thus, HCR-1 performs better in a dense network.

### 5.4. Transmission Latency Analysis

As we analyzed in the previous sections, HCR-1 uses relay selection in R(1) to balance the energy consumption and prolong the network lifetime. However, the relay selection in R(1) could lead to the growth of transmission latency. In this section, we use simulations to investigate the impacts of HCR-1 on the transmission latency. The hop count from the node to the sink is used as the metric to evaluate the transmission latency. In this scenario, the network area is set as 200 × 200 m^2^, and the number of nodes is 400. The clustering range $R_{c}$ is set as 70 m.

**Figure 12:**
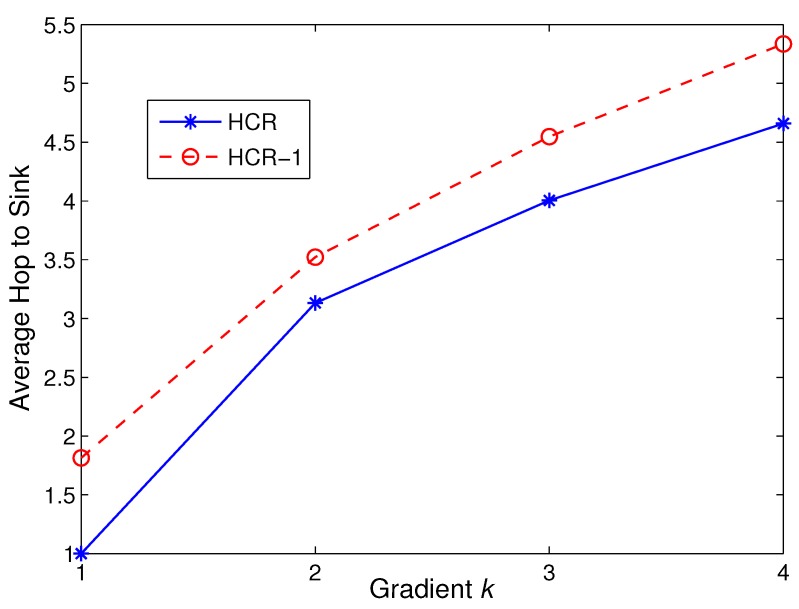
Average hop count comparison in different gradients.

The simulation results are classified by different gradients in Figure 12. As shown in Figure 12, the average hop counts in both HCR and HCR-1 have a tight relation to the gradient of the nodes. It is worth noting that the gradient of each node indicates the minimum transmission hop count from the node to the sink. In HCR, the average hop count is about k+1, which proves that HCR can generate a connected and sub-optimal topology, as analyzed in [19].

On the other hand, the average hop counts in HCR-1 are approximately 0.7 larger than those in HCR. This is because the inter-cluster routing algorithm used in R(1) adds one hop for the CHs with low residual energy (NEXT = 0) and their CMs. Nevertheless, the growth of hop counts in HCR-1 is tightly bound by one hop. Therefore, HCR-1 can improve the network lifetime with the reasonable growth of transmission latency.

## 6. Conclusions

In this paper, we study the energy consumption of HCR and discover an important result that HCR suffers from severe imbalanced energy consumption in R(1), *i.e.*, the nodes that can communicate with the sink directly. Based on this observation, we propose an improved protocol called HCR-1, which uses an adaptive energy threshold to classify nodes in R(1). Besides, the cost functions are designed to optimize the relay selection. A guideline for setting the parameters in HCR-1 is provided based on the simulations. The simulation results prove that HCR-1 effectively balances the energy consumption and prolongs the network lifetime by over 30% compared to that of HCR.
